# How organizational support drives knowledge worker performance: evidence for dual motivational and cognitive resource pathways

**DOI:** 10.3389/fpsyg.2026.1931481

**Published:** 2026-09-03

**Authors:** Xiaofang Xiong, Xiaofang Xiong

**Affiliations:** 1 Nanfang College, Guangzhou, China; 2 Suan Sunandha Rajabhat University, Bangkok, Thailand

**Keywords:** cognitive resources, concentration, dedication, job performance, motivational psychological state, perceived organizational support

## Abstract

**Introduction:**

Against the backdrop of the digital knowledge economy, insufficient motivation and sustained cognitive attention among knowledge workers relate to poor task performance. Drawing on social exchange theory and the Job Demands‑Resources (JD‑R) framework, this study investigated how two‑dimensional organizational support (instrumental work support and socio‑emotional value recognition) relates to Chinese knowledge workers’ job performance, with dedication (motivational resource) and concentration (cognitive attentional resource) as parallel mediators. Prior studies often adopt unidimensional perceived organizational support and undifferentiated work‑engagement constructs, overlooking separate motivational and cognitive pathways.

**Methods:**

Survey data from 346 Chinese knowledge workers were analyzed. We revised the Utrecht Work Engagement Scale by removing the redundant vigor dimension to create a knowledge‑work‑oriented two‑factor engagement scale. SPSS and AMOS were used for statistical analyses including confirmatory factor analysis, correlation, structural equation modeling, and Bootstrap mediation tests, with demographic variables controlled. All data are cross‑sectional and cannot confirm causality.

**Results:**

Both organizational‑support dimensions positively correlated with dedication, concentration, and job performance; work support yielded stronger cross‑sectional associations than value recognition. Dedication and concentration served as parallel partial correlational mediators, and concentration showed a marginally closer linkage with performance.

**Discussion:**

This study adds non‑Western localized correlational evidence for the JD‑R model by distinguishing motivational and cognitive resource pathways. It offers differentiated practical suggestions for optimizing workplace resources to sustain knowledge workers’ engagement. Future longitudinal research is needed to test causal relationships given cross‑sectional limitations.

## 1 Introduction

Existing organizational behavior research grounded in social exchange theory (SET) and perceived organizational support (POS) scholarship rests on a dominant yet under-theorized foundational assumption: organizational support is treated as a unitary resource that triggers homogeneous reciprocal responses among employees (Cropanzano and Mitchell, 2005). This oversimplified premise has become the mainstream empirical convention of single-indicator POS measurement, despite the Job Demands–Resources (JD-R) framework’s clear theoretical division between instrumental task resources and socio-emotional psychological resources (Berraies and Chouiref, 2023). Mainstream SET-POS research neglects this dimensional heterogeneity, assuming uniform psychological and behavioral outcomes from all supportive inputs. This study identifies this unexamined unified-resource assumption as the core source of theoretical distortion and inconsistent empirical findings across prior literature.

The JD-R framework draws an inherent categorical divide between instrumental and socio-emotional workplace resources, each activating distinct employee psychological processes (Bakker and Demerouti, 2008). Instrumental support removes operational barriers and eases cognitive burden by targeting employees’ cognitive processing systems, while symbolic value recognition fulfills self-esteem and belonging needs to drive affective motivation (Yadav et al., 2022). Complementary refined SET logic distinguishes two reciprocal mechanisms: tangible task resources elicit task-based cognitive reciprocity, while identity affirmation fosters identity-based motivational reciprocity (Bahadır et al., 2024). Conventional POS research conflates these two functionally independent resource categories into one latent construct, masking their divergent exchange and outcome effects and creating systematic theoretical averaging bias. Merging instrumental work support and socio-emotional value recognition erases their differentiated predictive strengths, blurs the resource-performance transmission chain, and yields conflicting POS-performance empirical results, limiting the explanatory power of POS theory for intellectual work contexts (Aboramadan and Karatepe, 2021; Novales et al., 2025).

This measurement and theoretical flaw is especially salient for knowledge worker research. Unlike standardized manual labor, knowledge value creation relies on prolonged deep cognition, creative problem-solving, and sustained learning, which demand separate motivational vitality and continuous attentional resources (Al-Omar et al., 2019). Existing work engagement measurement fails to fit such cognition-intensive work: the classic three-factor UWES was designed for general labor, with a physical stamina-focused vigor dimension that creates severe conceptual overlap with dedication’s intrinsic motivation; its original absorption subscale also cannot capture the long-duration high-intensity cognitive focus required by intellectual tasks (Rasool et al., 2021; Schaufeli et al., 2002; Wang, 2022). Few localized studies have developed and validated a motivation–cognition two-factor engagement structure tailored to knowledge workers, leaving the resource–engagement–performance pathway theoretically ambiguous and empirically underexplored.

Furthermore, Western-originated POS and JD-R frameworks lack sufficient contextual adaptability for Chinese knowledge practitioners. Built upon individualistic Western organizational norms, existing models are validated with general employee samples and fail to accommodate the high autonomy, extended cognitive cycles, and unique organizational exchange logics of domestic intellectual workers (Liu et al., 2024; Weer and Greenhaus, 2020). Sufficient localized correlational evidence remains absent, restricting scholars’ understanding of classic resource-exchange theories’ boundary conditions in non-Western digital work contexts.

To address these multilayered theoretical and contextual gaps, this study constructs a dual-resource, dual-engagement parallel mediation model rather than merely listing superficial dimensional defects. Its closed-loop research logic proceeds sequentially: flawed unified SET reciprocity assumptions → averaging bias from unidimensional POS measurement → ambiguous dual motivational-cognitive pathways in knowledge work → poor cross-cultural fit of Western theories → targeted theoretical revision and framework optimization. Specifically, differentiated exchange mechanisms of instrumental and emotional support are verified to revise SET’s generalized homogeneous reciprocity premise. The redundant UWES vigor dimension is eliminated, and dedication (motivation) and concentration (cognition) are separated as independent mediators to clarify how distinct organizational resources correlate with performance via targeted psychological states.

This study delivers three core theoretical innovations beyond scale revision and Chinese contextual supplementation. First, it revises the dominant unified reciprocity assumption in SET-POS research to resolve long-standing bias from undifferentiated resource modeling. Second, it disentangles intertwined motivational and cognitive engagement mechanisms to build a dual-transmission paradigm exclusive to cognitive-intensive knowledge labor. Third, it delineates heterogeneous functional boundaries between the two POS subdimensions, reconciling inconsistent prior POS-performance results and supplementing contextual boundary conditions for JD-R and SET theories.

Empirically, cross-sectional correlational pathways linking dual-dimensional support, two-factor engagement, and job performance are examined using a sample of 346 Chinese knowledge workers. The tentative correlational findings provide differentiated resource allocation guidance for knowledge-intensive enterprises, enabling managers to match organizational supplies with employees’ motivational and cognitive demands, stabilize sustained engagement, and maintain long-term innovative output amid intense industry talent competition (Bashir et al., 2020; Cao et al., 2019).

## 2 Literature review and hypotheses development

### 2.1 Perceived organizational support as a multidimensional resource

Perceived organizational support (POS) reflects employees’ holistic perception of organizational resource supply, effort recognition, and wellbeing care (Eisenberger et al., 1986). Rooted in organizational support theory, favorable organizational support fosters positive employee work attitudes and productive behaviors through reciprocal interaction mechanisms (Wang, 2022). Existing empirical studies have widely confirmed the positive association between POS and individual work outcomes, yet most prior studies treat POS as a single undifferentiated construct, ignoring its inherent resource heterogeneity and triggering theoretical averaging bias (Aboramadan and Karatepe, 2021).

This study’s two-dimensional decomposition of POS strictly follows the core resource classification logic of the JD-R model, which classifies all job resources into two mutually exclusive, heterogeneous categories: instrumental task resources and socio-emotional psychological resources (Bakker and Demerouti, 2008). This dichotomy provides the foundational theoretical premise to split POS into work support and value recognition, as the two subdimensions correspond exactly to JD-R’s dual resource typology. Complementarily, refined social exchange theory supplies micro-level psychological mechanisms to explain why the two POS dimensions produce distinct employee responses, rather than relying on the oversimplified homogeneous reciprocity assumption adopted in traditional POS research (Cropanzano and Mitchell, 2005).

Combined with the core resource classification logic of the JD-R framework, workplace supportive resources can be divided into two fundamentally distinct categories: instrumental task resources and socio-emotional psychological resources (Bakker and Demerouti, 2008). These two resources target different employee needs and initiate differentiated psychological activation processes, which is the core theoretical basis for dimensional decomposition of POS in this study. Specifically, instrumental work support refers to task-oriented tangible resources including work tools, technical support, professional training, and job autonomy, which directly eliminate operational and cognitive obstacles in task execution. In contrast, value recognition represents socio-emotional symbolic resources, such as intellectual achievement affirmation and professional identity respect, which satisfy employees’ psychological needs of self-esteem and organizational belonging rather than assisting specific task completion (Musenze et al., 2022; Nuutinen et al., 2022).

SET further clarifies the micro-psychological logic of differentiated resource effects. Unlike generalized macro reciprocal assumptions, the refined exchange mechanism in this study indicates that instrumental resources trigger task-based reciprocal cognition, whereby employees repay organizational instrumental support by improving work efficiency and cognitive input; socio-emotional resources stimulate identity-based reciprocal motivation, whereby employees reciprocate organizational emotional recognition with higher intrinsic work enthusiasm (Bahadır et al., 2024; Blau, 2017). This study distinguishes two-dimensional POS based on JD-R resource attributes and explains its differentiated psychological functions via SET micro-mechanisms, laying a solid theoretical foundation for subsequent pathway hypothesis derivation.

### 2.2 Differential effects of organizational support on motivational and cognitive resources

Matching the dual-resource theoretical framework established above, this study reconstructs the dimensional structure of work engagement to adapt to the cognitive–intensive nature of knowledge work. Unlike routine manual labor relying on physical operation, knowledge workers’ value creation depends on continuous analytical reasoning and creative thinking, which requires two independent endogenous psychological resources: intrinsic motivational willingness and sustained cognitive attention (Al-Omar et al., 2019). The traditional three-factor UWES scale is poorly applicable to intellectual labor: its physical-stamina-based vigor dimension presents severe conceptual overlap with dedication’s intrinsic motivational attributes, causing measurement redundancy. Meanwhile, the original absorption dimension only reflects general work immersion and fails to capture the high-intensity, long-duration cognitive focus required by complex knowledge tasks. Accordingly, this study eliminates the redundant vigor dimension and redefines absorption as concentration, forming a refined two-factor engagement structure consisting of motivational dedication and cognitive concentration (Chen et al., 2020; Wang, 2022).

The original three-factor UWES was developed for general labor groups with physically intensive work characteristics, and its vigor dimension operationalizes energy as physical stamina, a construct that is less relevant to the core work process of knowledge workers (Schaufeli et al., 2002). For intellectual labor that relies on long-cycle deep thinking, the intrinsic motivational enthusiasm captured by dedication fully covers the positive energy connotation relevant to knowledge work, making the physical stamina-focused vigor dimension redundant and creating serious conceptual overlap between vigor and dedication (Xu et al., 2025). Moreover, the original absorption subscale only captures transient general work immersion and fails to operationalize the sustained, high-intensity cognitive concentration required for complex knowledge creation tasks.

This study adopts an integrated JD-R and SET theoretical system to explain the differentiated resource activation mechanisms. From the JD-R perspective, instrumental work support and socio-emotional value recognition represent heterogeneous workplace resources with distinct functional paths (Bakker and Demerouti, 2008). Instrumental work support provides task-facilitating resources that eliminate operational obstacles and reduce cognitive load, allowing knowledge workers to concentrate limited psychological resources on core analytical and innovative tasks and thereby enhance cognitive concentration. In contrast, value recognition satisfies employees’ identity and belonging needs, strengthens emotional identification with organizational goals, and effectively stimulates intrinsic work dedication.

Refined SET micro-psychological mechanisms further underpin these differentiated effects through two reciprocal logics (Azeem et al., 2021). Instrumental resource supply beyond contractual obligations fosters equitable task exchange perceptions, triggering task-based cognitive reciprocity, in which employees repay organizational resource support with sustained focused cognitive investment. By contrast, continuous organizational value recognition builds high-quality long-term social exchange bonds, forming identity-based motivational reciprocity that promotes voluntary emotional devotion and work dedication. This organic integration avoids fragmented theoretical justification and forms a cohesive logical chain for resource-induced engagement activation.

Based on the above systematic theoretical deduction, this study proposes the following hypotheses, which are fully consistent with the empirical model in subsequent analysis:

*H1a*: Work support is positively related to dedication (motivational resource).

*H1b*: Work support is positively related to concentration (cognitive attentional resource).

*H1c*: Value recognition is positively related to dedication (motivational resource).

*H1d*: Value recognition is positively related to concentration (cognitive attentional resource).

### 2.3 Work engagement and job performance

Knowledge work differs substantially from traditional routine labor, as individual innovation and task accomplishment depend on prolonged complex cognition, independent analytical reasoning, and iterative creative problem-solving (Vuori et al., 2019). These unique occupational characteristics mean that knowledge workers’ performance is highly sensitive to internal psychological resource states rather than merely generalized work motivation. However, prior literature generally operationalizes work engagement as a holistic construct without separating motivational and cognitive sub-processes. This conventional undifferentiated treatment obscures their distinct predictive mechanisms and results in oversimplified theoretical explanations regarding how engagement translates into superior job performance (Weer and Greenhaus, 2020).

Consistent with the integrated JD-R and SET theoretical framework of this study, the refined dual-dimensional engagement structure enables a precise interpretation of differentiated performance pathways. As the motivational component of engagement, dedication reflects employees’ intrinsic enthusiasm, organizational identification, and affective commitment to work goals. Triggered by positive social exchange reciprocity, dedicated employees exhibit proactive work investment, voluntary extra -role behaviors, and persistent task involvement, which comprehensively improve overall task quality and job performance (Rasool et al., 2021; Yadav et al., 2022). As the cognitive component, concentration captures sustained deep immersion and focused attention during knowledge-intensive tasks. Continuous cognitive focus reduces attentional distraction and resource switching loss, ensuring rigorous information processing, innovative thinking, and high-standard task delivery that constitute the core cognitive foundation of knowledge workers’ performance advantages (Bäcklander and Richter, 2022).

Distinguishing dedication and concentration addresses the theoretical limitation of unified engagement modeling. It establishes two independent yet complementary psychological pathways: motivational dedication promotes performance through affective behavioral devotion, while cognitive concentration facilitates performance through efficient attentional resource utilization and high-quality cognitive processing. This differentiated resource transformation logic coheres closely with the dual-resource activation mechanism in the previous section, forming a complete and self-consistent theoretical chain that explains how internal psychological resources mediate the linkage between external organizational support and individual job performance.

Given the above theoretical reasoning, this study puts forward two predictive hypotheses:

*H2a*: Dedication is positively related to job performance.

*H2b*: Concentration is positively related to job performance.

### 2.4 The mediating roles of dedication and concentration

This section adopts a cohesive two-layer integration of the JD-R framework and social exchange theory (SET) to interpret the mediating mechanism, avoiding the *ad hoc* theoretical stitching common in prior studies. Within this unified system, JD-R theory provides structural classification logic, distinguishing instrumental and socio-emotional organizational resources and positioning external support as a key exogenous driver of employees’ internal motivational and cognitive resources (Bakker and Demerouti, 2008). In turn, SET offers micro-psychological explanations, clarifying how differentiated resource inputs trigger distinct reciprocal processes and translate situational organizational support into individual behavioral and performance outcomes (Cropanzano and Mitchell, 2005).

For cognition-intensive knowledge work, organizational support exerts indirect performance effects rather than direct influences, operating through the activation of internal psychological resources. Instrumental work support removes task obstacles and reduces cognitive consumption, fostering sustained cognitive concentration via task-based reciprocal exchange. In contrast, value recognition satisfies employees’ identity and belonging needs, strengthens organizational attachment, and boosts intrinsic dedication through identity-based reciprocal mechanisms (Mingione and Leoni, 2020). Collectively, dual-dimensional organizational support elevates motivational and cognitive engagement, which serve as core intermediate psychological carriers linking external resources to job outcomes.

**Figure 1:**
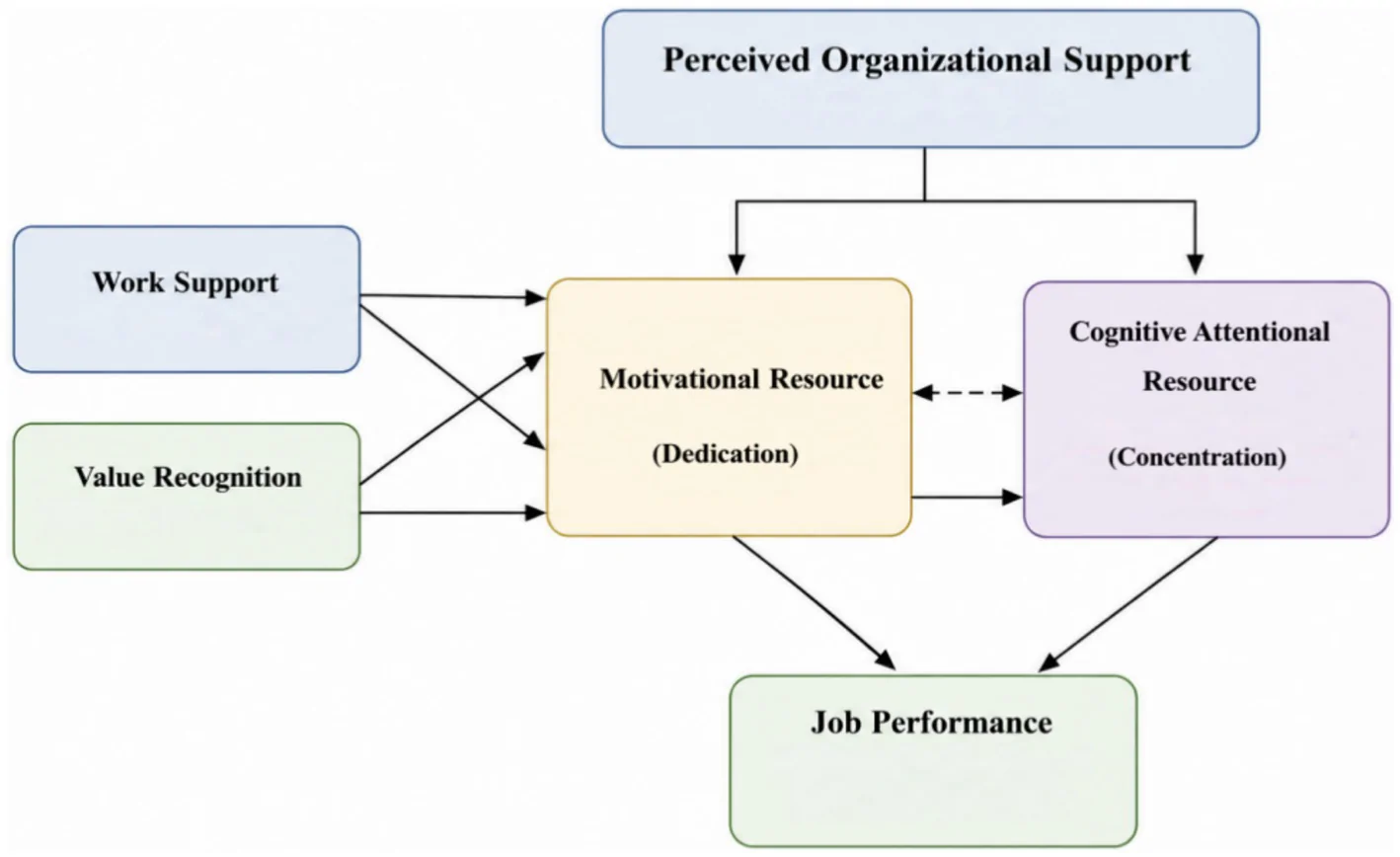
Proposed conceptual model.

Improved dedication and concentration further promote superior task performance. Dedication facilitates proactive work investment and voluntary engagement, while concentration guarantees efficient information processing and high-quality innovative problem-solving. As parallel mediating variables, dual engagement states convert heterogeneous organizational resource supply into stable performance advantages among knowledge workers (Bahadır et al., 2024; Nuutinen et al., 2022). This multi-path mediation mechanism integrates the differentiated resource activation logic from prior sections and forms a complete theoretical chain for the research model (Figure 1).

Given the above theoretical deduction, this study puts forward four partial mediation hypotheses:

*H3a*: Dedication mediates the relationship between work support and job performance.

*H3b*: Dedication mediates the relationship between value recognition and job performance.

*H3c*: Concentration mediates the relationship between work support and job performance.

*H3d*: Concentration mediates the relationship between value recognition and job performance.

### 2.5 Definition of knowledge workers

Consistent with Drucker’s classic definition, knowledge workers refer to employees whose core job responsibilities revolve around the creation, analysis, application, and dissemination of professional knowledge, instead of repetitive standardized manual labor (Drucker, 2001). Such employees must master specialized expertise, possess independent problem-solving judgment, and sustain lifelong learning to fulfill work demands.

To match this definition, the present study adopts targeted sampling covering typical knowledge-intensive industries: information technology, R&D, finance, consulting, and management roles that demand professional expertise and independent decision-making authority. All participants are full-time practitioners engaged in intellectual labor rather than routine service or production posts. These sectors are universally acknowledged in prior literature as representative samples of knowledge workers.

## 3 Methodology

### 3.1 Sample and data collection

Data were collected over 28 days (1–28 April 2025) via online convenience sampling using the Credamo platform and industry WeChat communities. Invalid responses were screened out based on completion duration, repeated scoring, blank core items, and logical contradictions. The final sample consisted of 346 valid questionnaires, with an effective response rate of 87.15%.

Participants were full-time employees from knowledge-intensive sectors, including information technology, R&D, finance, consulting, and management. Their work features knowledge creation, independent analysis, and autonomous decision-making, which conforms to the characteristics of knowledge workers and provides a suitable context for model validation.

The sample presents a skewed age distribution, with 57.2% of respondents aged under 30 and 89.6% under 40. This pattern reflects the practical workforce structure of knowledge-intensive industries, where young employees with high digital adaptability and learning competence dominate cognitive and innovative work. While the sample matches the industry context, the unbalanced age structure may limit representativeness. Future studies can adopt stratified age sampling to enhance generalizability. Detailed demographic statistics are shown in Table 1.

**Table 1:** Demographic characteristics.

Characteristic	Category	Frequency	Percentage
Gender	Male	182	52.6%
	Female	164	47.4%
Age	≤30 years	198	57.2%
	31–40 years	112	32.4%
	41–50 years	28	8.1%
	≥51 years	8	2.3%
Education	Bachelor’s degree	210	60.7%
	Master’s degree or higher	136	39.3%
Work experience	<3 years	98	28.3%
	3–7 years	156	45.1%
	>7 years	92	26.6%
Industry	IT/Technology	124	35.8%
	Finance	86	24.9%
	Consulting	52	15.0%
	R&D	48	13.9%
	Other	36	10.4%

The sample overrepresents young employees aged 35 and below (approximately 68% of total participants). This skewed age composition is acknowledged as one key limitation affecting the generalizability of findings.

This study adopts single-source cross-sectional self-report data, which may cause common method bias (CMB). To alleviate this problem, procedural controls including anonymous survey participation, randomized item arrangement, and validated scale adoption were applied. Harman’s single-factor test confirmed that CMB did not substantially affect data validity.

### 3.2 Variable measurement

This study measures three core latent constructs: perceived organizational support (POS), work engagement, and job performance. The questionnaire is split into two modules. The first module collects demographic control variables: gender, age, education, corporate type, job position, and organizational tenure. The second module contains validated measurement items for the three focal constructs. All items adopt a five-point Likert agreement scale (1 = strongly disagree, 5 = strongly agree). Every scale selected in this paper has been repeatedly verified for robust reliability and validity in existing domestic and international empirical research.

#### 3.2.1 Perceived organizational support (independent variable)

POS is measured via the two-dimensional scale validated by Dianingrum and Hifdhillah (2021). This instrument has been widely adopted in Chinese organizational behavior research with consistently satisfactory psychometric outcomes. It contains 10 items split evenly across two subdimensions: five items for work support (instrumental task resources) and five items for value recognition (socio-emotional affirmation). The original ambiguous term “job support/value identity” is standardized uniformly as work support and value recognition throughout the manuscript.

#### 3.2.2 Work engagement (mediator variable)

Adapting the classic Utrecht Work Engagement Scale (Schaufeli et al., 2002) and localized research targeting Chinese knowledge workers, the vigor dimension is eliminated, and the original absorption subscale is redefined as concentration to capture sustained deep cognitive focus inherent to intellectual labor, yielding a refined two-factor measurement with four dedication items measuring motivational resources and five concentration items capturing cognitive attentional resources. Separate confirmatory factor analyses (CFAs) are executed on identical engagement item datasets to quantitatively contrast model fit between the original three-factor UWES and the revised two-factor structure. Comparative fit statistics are presented in Table 2.

**Table 2:** Comparative CFA fit: original 3-factor vs. revised 2-factor Work Engagement Scale.

Fit index	Threshold for good fit	Original 3-factor UWES	Revised two-factor scale
*χ* ^2^	–	267.812	136.407
df	–	62	34
*χ*^2^/df	<3	4.320	2.071
CFI	>0.90	0.902	0.968
TLI	>0.90	0.879	0.956
RMSEA	<0.08	0.103	0.055
SRMR	<0.08	0.091	0.042

As illustrated in Table 2, the original three-factor UWES fails to meet acceptable fit standards (*χ*^2^/df > 3, RMSEA & SRMR > 0.08), while the revised two-factor model satisfies all ideal fit criteria. Multiple psychometric validations, such as confirmatory factor analysis, reliability assessment, and convergent validity verification, confirm superior model fit and structural suitability of the two-factor structure for knowledge workers relative to the original three-factor UWES, with all items and dimensional indices meeting rigorous psychometric standards to guarantee reliable and stable measurement within the current research context.

#### 3.2.3 Job performance (dependent variable)

Job performance is measured using the four-item scale developed by Chiang and Hsieh (2012). Representative sample items include “I can complete all assigned work tasks on schedule” and “The quality of my output meets official organizational standards.” This scale integrates overall work performance without separating task performance and contextual performance, which is noted as a measurement limitation in Section 5.5.

### 3.3 Data analysis methods

All statistical analyses were performed via SPSS 24.0 and AMOS 24.0, following four sequential analytical procedures.

1. Scale reliability and construct validity tests: Cronbach’s *α*, CITC, CFA (convergent validity: CR, AVE; discriminant validity: Fornell–Larcker criterion).
2. Pearson correlation analysis for pairwise relationships between latent variables.
3. Structural equation modeling (SEM) to test direct paths; model fit indices: *χ*^2^/df, CFI, TLI, NFI, RMSEA, SRMR.
4. Control variable processing: gender, age, industry, and organizational tenure were set as exogenous control variables predicting job performance simultaneously with core paths. Control variable coefficients are placed in appendix tables for brevity. All control variables showed insignificant effects on job performance (*p* > 0.05).

### 3.4 Ethical statement

This research obtained formal ethical approval from the Ethics Committee of Suan Sunandha Rajabhat University (Approval Code: 67-278-2-1; COE.2-286/2025). All survey participants provided voluntary digital informed consent by checking a mandatory consent checkbox at the start of the online questionnaire before accessing formal measurement items. The survey strictly adopted a full anonymization design: no real names, employee IDs, company information, or identifiable personal background information were collected. All completed questionnaire data were encrypted and stored offline, and only aggregated statistical results were used for subsequent analysis to eliminate privacy risks. The whole data collection and analysis procedure fully complied with institutional ethical guidelines and the core principles outlined in the Declaration of Helsinki.

## 4 Empirical analysis

### 4.1 Reliability analysis

In this research, SPSS 24.0 was employed to evaluate the reliability of the gathered data (Table 3). The Cronbach’s *α* value for the five subscales exceeded 0.8, and all item-level Corrected Item-Total Correlation (CITC) values in this study were above 0.4. Furthermore, removing any individual question did not result in an increase in the Cronbach’s *α* value, suggesting that the five subscales demonstrate strong reliability.

**Table 3:** Scale reliability analysis.

Variable	Dimension	Item	CITC	Cronbach’s *α* value after deleting items	Cronbach’s *α* value
Perceived organizational support	Work support	WS1	0.777	0.918	0.928
		WS2	0.827	0.908	
		WS3	0.783	0.917	
		WS4	0.826	0.909	
		WS5	0.840	0.906	
	Value recognition	VR1	0.815	0.929	0.939
		VR2	0.876	0.917	
		VR3	0.881	0.916	
		VR4	0.841	0.924	
		VR5	0.773	0.937	
Work engagement	Dedication	DED1	0.754	0.759	0.839
		DED2	0.642	0.813	
		DED3	0.680	0.793	
		DED4	0.625	0.817	
	Concentration	CON1	0.784	0.873	0.901
		CON2	0.719	0.887	
		CON3	0.781	0.874	
		CON4	0.774	0.875	
		CON5	0.715	0.888	
Job performance		JP1	0.720	0.840	0.873
		JP2	0.779	0.816	
		JP3	0.742	0.831	
		JP4	0.670	0.860	

### 4.2 Validity analysis

Factorability of the data was examined using the Kaiser–Meyer–Olkin (KMO) index and Bartlett’s test of sphericity. A KMO score greater than 0.70 and a significant Bartlett’s test result (*p* < 0.05) provided strong evidence for the adequacy of the sample for factor analytic procedures. As shown in Table 4.

**Table 4:** KMO and Bartlett’s test of sphericity.

KMO value		0.932
Bartlett sphericity test	Approximate chi-square	4,879.306
	Df	171
	*p*-value	0.000

The scale’s construct validity was verified using Confirmatory Factor Analysis techniques, which determines how well the measured indicators reflect their corresponding latent constructs. The results of the validation factor analysis are shown in Table 5.

**Table 5:** Validated factor analysis.

Variable	Item	Factor loadings	AVE	CR
Work support	WS1	0.815	0.722	0.928
	WS2	0.882		
	WS3	0.867		
	WS4	0.817		
	WS5	0.864		
Value recognition	VR1	0.847	0.759	0.940
	VR2	0.918		
	VR3	0.925		
	VR4	0.865		
	VR5	0.795		
Dedication	DED1	0.889	0.573	0.842
	DED2	0.681		
	DED3	0.717		
	DED4	0.725		
Concentration	CON1	0.836	0.647	0.901
	CON2	0.758		
	CON3	0.842		
	CON4	0.829		
	CON5	0.751		
Job performance	JP1	0.777	0.635	0.874
	JP2	0.863		
	JP3	0.823		
	JP4	0.718		

As shown in the table, all five latent variables—work support, value recognition, dedication, concentration, and job performance—exhibit factor loadings above 0.60. Additionally, all composite reliability (CR) values exceed 0.70, and all average variance extracted (AVE) values are higher than 0.50, indicating that the scale meets the required thresholds for convergent validity.

Discriminant validity was assessed using established criteria. Specifically, it is confirmed when the square root of a construct’s AVE exceeds its correlations with any other construct, demonstrating that each construct maintains a unique identity within the measurement model. As shown in Table 6.

**Table 6:** Distinguishing validity and correlation coefficients.

Variable	Work support	Value recognition	Dedication	Concentration	Job performance
Work support	**0.850**				
Value recognition	0.292	**0.871**			
Dedication	0.572	0.445	**0.757**		
Concentration	0.484	0.386	0.552	**0.804**	
Job performance	0.485	0.373	0.519	0.702	**0.786**

Bolded values are the AVE open root value of the variables.

The tabulated results demonstrate robust discriminant validity across the measured constructs. Based on the outcomes of preceding statistical assessments, the survey instrument employed in this study satisfies the required standards for both reliability and validity. Consequently, the dataset obtained is deemed appropriate for conducting structural equation modeling and subsequent hypothesis testing.

### 4.3 Common method bias test

Since all data were collected via self-reported questionnaires from a single source, common method bias (CMB) constitutes a potential confounding risk. This study implemented multiple procedural controls at the questionnaire design and distribution stage to suppress method artifacts in advance. First, all respondents were guaranteed full anonymity, with no personally identifiable information collected to reduce social desirability bias. Second, items measuring different core constructs were randomly reordered and separated into distinct questionnaire modules to avoid consistent response sets. Third, several reverse-coded items were interspersed among positive measurement statements to prevent mechanical uniform scoring by participants.

Statistical double verification was further conducted to quantitatively examine CMB. First, Harman’s single-factor test was adopted: all measurement items were loaded into an unrotated exploratory factor analysis. The first extracted factor only explained 32.84% of total variance, falling below the 50% critical threshold for severe common method variance. Beyond Harman’s single-factor test, this study further adopted the unmeasured latent method factor approach to mitigate common method bias concerns. We constructed an alternative CFA model that incorporated a global method latent variable loading all survey items alongside substantive theoretical constructs. Model comparison results indicated negligible changes to standardized path coefficients and overall model fit after adding the method latent variable. No substantial shifts in construct correlations or structural paths were detected between the baseline measurement model and the method-factor-integrated model, which confirms common method variance does not substantially distort the correlational patterns observed in this sample. Taken together, procedural controls and dual statistical tests jointly rule out serious CMB interference.

### 4.4 Correlation analysis

Table 7 presents the means, standard deviations, and Pearson correlation coefficients for all core study variables. The results show that work support, value recognition, dedication, concentration, and job performance are significantly and positively intercorrelated (*p* < 0.01). Specifically, the two dimensions of perceived organizational support are positively associated with work engagement (dedication and concentration) and job performance. Likewise, both dedication and concentration demonstrate significant positive correlations with job performance.

**Table 7:** Results of correlation analysis of variables.

Variable	Work support	Value recognition	Dedication	Concentration	Job performance
Work support	1				
Value recognition	0.292**	1			
Dedication	0.576**	0.445**	1		
Concentration	0.484**	0.386**	0.549**	1	
Job performance	0.485**	0.373**	0.528**	0.497**	1

**represents statistical significance at the *p* < 0.01 level.

Correlation results further reveal that work support and value recognition are significantly correlated with job performance (*r* = 0.485, *p* < 0.01; *r* = 0.373, *p* < 0.01, respectively). The two POS dimensions also exhibit strong positive associations with dedication and concentration, with coefficients ranging from 0.386 to 0.576. In addition, dedication and concentration are positively correlated with job performance (*r* = 0.528 and *r* = 0.497, *p* < 0.01).

Notably, these bivariate correlations only provide preliminary descriptive evidence for the pairwise variable relationships proposed in the research hypotheses (H1a–H1d, H2a–H2b, H3a–H3d) and cannot serve as formal hypothesis validation. Correlation analysis does not account for structural covariation, pathway mechanisms, or mediating effects. Consequently, rigorous hypothesis testing and definitive empirical support for all hypothesized paths rely entirely on the subsequent structural equation modeling and Bootstrap mediation analyses reported in the following sections. Overall, the observed correlational patterns are consistent with the theoretical direction of the proposed model and lay a foundational statistical basis for further structural path analysis.

### 4.5 Direct effect analysis

**Figure 2:**
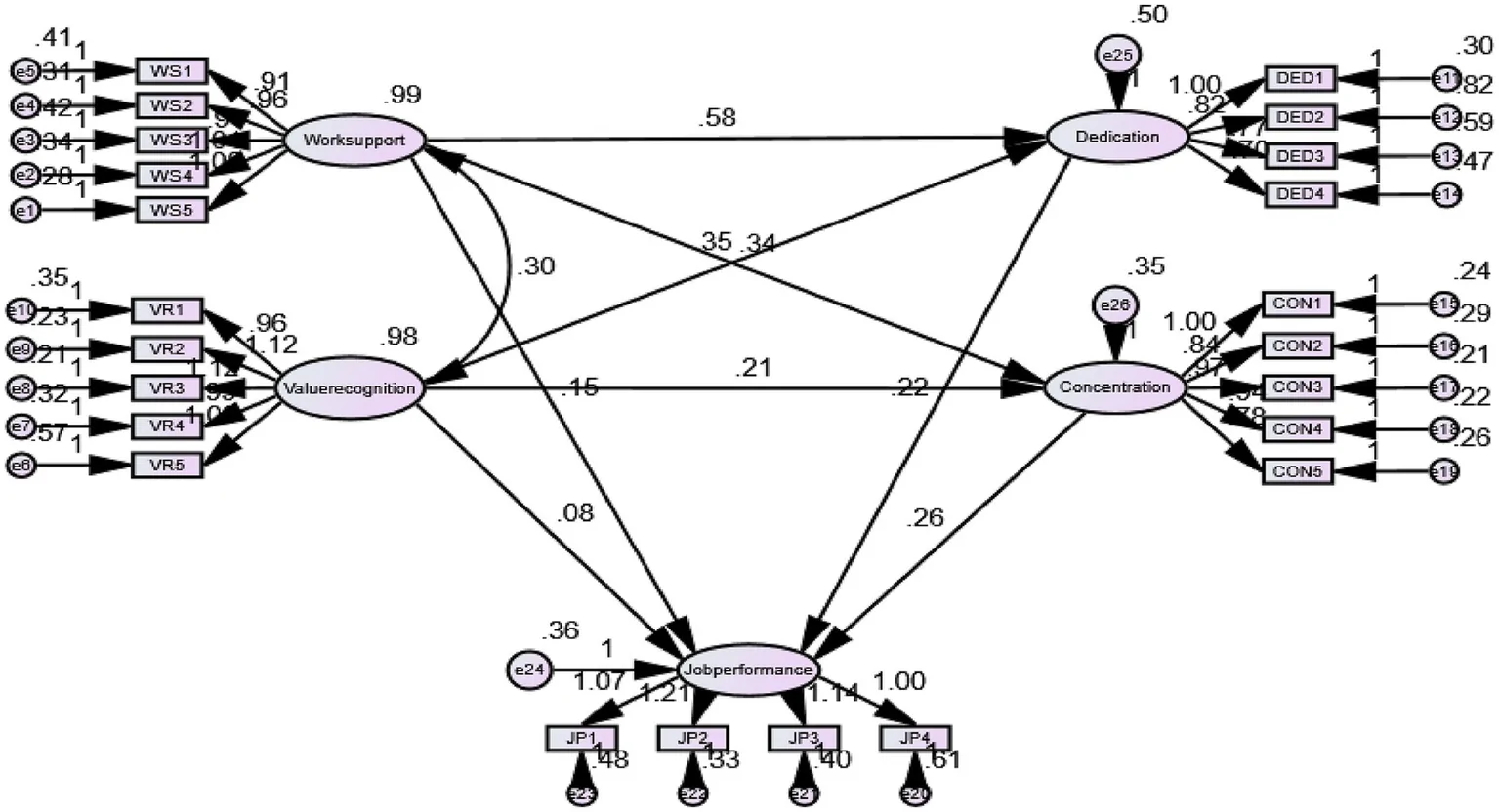
Structural equation model diagram.

In line with the proposed theoretical relationships, AMOS 24.0 was used to construct the structural equation model; paths with *p* < 0.05 were regarded as statistically significant. The structural model diagram is displayed in Figure 2.

Before interpreting path coefficients and testing hypotheses, model fit evaluation was conducted as a prerequisite. Only models satisfying standard fit criteria can support subsequent hypothesis verification; otherwise, structural adjustments are required. All fit indices are summarized in Table 8.

**Table 8:** Model fitness.

Model fitness metrics	Fitness value	Good fit	Acceptable fit	Results
*χ* ^2^	290.228			
Df	221			
*χ*^2^/df	1.313	<3	<5	Good fit
GFI	0.933	>0.90	>0.80	Good fit
AGFI	0.916	>0.90	>0.80	Good fit
NFI	0.951	>0.90	>0.80	Good fit
RFI	0.944	>0.90	>0.80	Good fit
IFI	0.988	>0.90	>0.80	Good fit
TLI	0.986	>0.90	>0.80	Good fit
CFI	0.988	>0.90	>0.80	Good fit
PNFI	0.831	>0.70	>0.60	Good fit
PCFI	0.863	>0.70	>0.60	Good fit
RMSEA	0.030	<0.08	<0.1	Good fit

All model fit indicators reach ideal thresholds: *χ*^2^/df = 1.313 (< 3), RMSEA = 0.030 (<0.08); GFI, AGFI, NFI, RFI, IFI, TLI and CFI all exceed 0.90; PNFI and PCFI are both above 0.70. The structural model exhibits satisfactory fit and is suitable for subsequent hypothesis testing. Standardized and unstandardized path coefficients are presented in Table 9.

**Table 9:** Path coefficient test table.

Path relationship	Unstandardized coefficient	Standardized coefficient	Standard deviation	*T*-value	*p*-value
Work support → job performance	0.166	0.218	0.043	3.871	***
Value recognition → job performance	0.092	0.130	0.035	2.598	0.009
Work support → dedication	0.364	0.484	0.032	11.216	***
Work support → concentration	0.290	0.406	0.034	8.638	***
Value recognition → dedication	0.214	0.303	0.030	7.033	***
Value recognition → concentration	0.179	0.267	0.031	5.685	***
Dedication → job performance	0.218	0.216	0.056	3.866	***
Concentration → job performance	0.240	0.227	0.054	4.419	***

*** represents significance at *p* less than 0.01.

Structural path results reveal significant positive cross-sectional correlations between both organizational support subdimensions and job performance. Work support exhibits a stronger statistical association with performance (*β* = 0.218, *p* < 0.001) than value recognition (*β* = 0.130, *p* = 0.009), indicating instrumental task resources have tighter concurrent linkages to knowledge workers’ task outcomes. No causal inferences can be drawn from these snapshot associations.

Work support also correlates more strongly with dedication (*β* = 0.484, *p* < 0.001) and concentration (*β* = 0.406, *p* < 0.001) than value recognition (*β* = 0.303; *β* = 0.267). This pattern only demonstrates stronger statistical co-occurrence between tangible workplace resources and employees’ motivational/cognitive engagement; cross-sectional data cannot verify causal promotion effects of work support.

Both dedication (*β* = 0.216, *p* < 0.001) and concentration (*β* = 0.227, *p* < 0.001) are positively correlated with job performance, with concentration showing a marginally larger coefficient. This merely reflects closer concurrent linkage between sustained deep cognitive focus and task performance rather than proving concentration causally improves work outcomes.

### 4.6 Mediation effect test

Bootstrap resampling with 2,000 iterations and bias-corrected 95% confidence intervals (CIs) was adopted to assess the indirect correlational pathways between variables. An effect is statistically significant when its CI excludes zero. If both the direct and indirect pathways have significant CIs, partial correlational mediation is identified; full mediation is indicated only when the direct path’s CI contains zero while the indirect path remains significant. All estimated total, direct, and indirect correlational values are summarized in Table 10.

**Table 10:** Mediating effects test table.

Type of effect	Variable relationship	Effect value	Bootstrap confidence interval	
			Lower	Upper
Total effect	Work support → job performance	0.315	0.243	0.386
	Value recognition → job performance	0.182	0.115	0.248
Direct effect	Work support → job performance	0.165	0.085	0.246
	Value recognition→ job performance	0.092	0.024	0.112
Indirect effect	Work support → dedication → job performance	0.079	0.043	0.167
	Work support → concentration → job performance	0.070	0.051	0.134
	Value recognition → dedication → job performance	0.047	0.024	0.112
	Value recognition → concentration → job performance	0.043	0.029	0.096

Within this cross-sectional sample, work support exhibits a statistically significant total correlational linkage with job performance (CI excludes zero). Its direct correlational association with job performance also remains significant, meaning work support is concurrently correlated with job performance both directly and through intermediate variables. Supporting the correlational logic of H3a, the indirect path via dedication carries a significant non-zero CI, confirming dedication acts as a partial correlational mediator. Likewise, the indirect path through concentration is statistically meaningful, consistent with the correlational pattern hypothesized in H3c.

Value recognition also shows a significant total correlational association with job performance, with its direct correlation retaining statistical significance after introducing the two engagement dimensions as intermediate variables. The indirect pathway passing through dedication yields a significant CI, matching the correlational expectation of H3b. Meanwhile, concentration also presents a meaningful indirect correlational linkage, providing sample evidence consistent with H3d.

Overall, the correlational patterns observed in this cross-sectional dataset align with all four mediation hypotheses (H3a, H3b, H3c, and H3d). The results reflect that dedication and concentration coexist as parallel partial correlational intermediates linking the two dimensions of perceived organizational support to job performance in the sampled group of knowledge workers.

## 5 Results and discussion

### 5.1 Results

All results merely capture snapshot cross-sectional correlations, with no causal, directional, or temporal inferences permissible from this single-wave dataset. SEM and Bootstrap analyses reveal three core correlational patterns. First, work support correlates more strongly with dedication and concentration than value recognition, showing tighter statistical co-occurrence between instrumental resources and employees’ motivational/cognitive engagement. Second, both dedication and concentration positively correlate with job performance; concentration’s coefficient is marginally larger (difference = 0.011), with no meaningful disparity in their correlational magnitudes. Third, dedication and concentration act as parallel partial correlational intermediates linking the two POS subdimensions to performance.

Collectively, organizational support exhibits dual concurrent associations with job performance: direct correlations plus indirect correlational pathways via the two engagement dimensions. These patterns align with the study’s theoretical directional expectations and offer tentative cross-sectional evidence of heterogeneous resource correlations. Longitudinal research is necessary to validate potential causal sequences.

### 5.2 Discussion

This study develops a refined parallel mediation model by splitting POS and separating work engagement into motivational and cognitive dimensions. The correlational gap between work support and value recognition matches JD-R’s instrumental/symbolic resource dichotomy and resolves inconsistent POS-performance findings in prior studies. Compared with Western general employee samples, Chinese knowledge workers exhibit stronger concurrent links between tangible task resources and dual engagement states—a rarely noted cultural trait. The two-factor engagement scale achieves better fit than the physical-focused original UWES, as dedication and concentration capture intellectual labor’s dual psychological needs. Beyond hypothesis testing, these correlational results deliver localized insights and address oversimplified Western theoretical frameworks.

First, the differential predictive effects of work support and value recognition advance the dimensional refinement of POS theory. Traditional organizational support research mostly treats POS as a unified construct, failing to distinguish the heterogeneous functional values of instrumental resources and socio-emotional care (Al-Omar et al., 2019; Wang, 2022). This study confirms that tangible work support serves as a more practical and effective organizational resource for knowledge workers’ engagement and performance improvement. For intellectual labor dominated by knowledge creation, data analysis, and innovative problem-solving, operational resources including technical tools, professional training, and job autonomy can directly reduce work barriers and optimize task execution efficiency (Goyal et al., 2020). In contrast, value recognition mainly satisfies employees’ emotional and belonging needs, which exerts limited immediate improvement on task performance (Mingione and Leoni, 2020). This outcome clarifies the differentiated functional mechanisms of dual-dimensional POS, revises the single-dimensional cognition of organizational support effectiveness, and explains why instrumental resource support generates more practical work value in high-cognitive-intensity work scenarios.

Second, the equivalent predictive importance of dedication and concentration supplements the localized boundary conditions of work engagement theory. Existing classic engagement frameworks mostly adopt a unified three-factor structure centered on general employees (Berraies and Chouiref, 2023), lacking targeted applicability for knowledge workers with long-term deep cognitive work characteristics. This study verifies that motivational dedication and cognitive concentration jointly constitute the core psychological driving forces of knowledge workers’ high performance. Although concentration shows a slightly higher coefficient, the two dimensions exhibit essentially equal practical value, indicating that sustainable motivational investment and continuous cognitive focus are irreplaceable complementary conditions for high-quality intellectual work. This finding corrects the potential bias of overemphasizing cognitive focus alone and reveals the unique dual-psychological resource operating mechanism of Chinese knowledge workers, which enriches the contextual adaptability of Western classic engagement theories in Eastern innovation-oriented industries.

Third, the verified parallel partial mediation mechanism expands the multi-path theoretical framework of “organizational support-work state-work outcome”. The coexistence of significant parallel mediation effects and residual direct effects indicates that organizational support influences job performance through both explicit motivational-cognitive engagement pathways and implicit unmeasured psychological channels (such as self-efficacy and psychological safety). The majority of previous studies adopt single-mediator linear models, which obscure the multi-dimensional psychological transmission mechanism of organizational resources (Bahadır et al., 2024; Musenze et al., 2022; Rasool et al., 2021). By decomposing POS and work engagement into fine-grained sub-constructs, this study constructs a more precise explanatory framework, reveals the multi-layered influencing logic of organizational resources on intellectual work performance, and provides a new analytical perspective for subsequent refined research on knowledge worker management.

Notably, the core findings of this study are constrained by inherent research design limitations and possess clear applicable boundaries. The cross-sectional self-report design cannot establish causal temporal sequences between variables, leaving potential reverse explanatory relationships unaddressed. Meanwhile, the young-dominated sample structure adapts to the talent characteristics of emerging knowledge-intensive industries but limits the generalizability of the conclusions to older practitioners and traditional industry groups. Future research can adopt longitudinal tracking designs to verify causal relationships and employ stratified sampling to optimize sample representativeness, so as to further improve the robustness and universality of the multi-path theoretical framework proposed in this study.

### 5.3 Theoretical implications

Against the backdrop of China’s digital economy, traditional Western organizational behavior theories, established for routine labor, cannot fully explain how modern knowledge workers convert organizational resources into job performance. Addressing this gap, this study presents three cohesive theoretical advancements that refine classic theories and close the research loop for digital-economy talent management.

First, this study advances a context-differentiated organizational support mechanism. Departing from the conventional unidimensional view of perceived organizational support, it verifies that instrumental work support more effectively facilitates knowledge workers’ engagement and performance than socio-emotional value recognition. This finding revises the generalized effectiveness assumption of classic POS theory, clarifies its digital-industry boundary conditions, and enriches localized resource allocation theories for high-cognitive work scenarios.

Second, this study optimizes the structural dimensionality of work engagement for digital intellectual labor. The original UWES framework, which emphasizes physical vigor, is ill-suited to sustained cognitive work. By removing the vigor dimension and adopting a dedication–concentration dual-factor structure, this study revises traditional engagement measurement, aligns the theoretical framework with knowledge workers’ cognitive working features, and improves its contextual adaptability in digital innovation scenarios.

Third, this study extends JD-R and social exchange theory via a localized parallel mediation perspective. Unlike Western single-path models, this study reveals dual motivational and cognitive psychological pathways linking organizational support to job performance. It establishes a complete “resource–engagement–performance” explanatory chain for Chinese knowledge workers, remedies the contextual limitations of generalized Western theories, and provides a refined theoretical paradigm for digital talent management research.

### 5.4 Practical implications

From the perspective of employee psychological resource maintenance, cognitive attention protection, and intrinsic motivation activation, firms should prioritize instrumental work support to ease cognitive depletion, paired with value recognition to sustain internal motivation, forming a dual psychological resource protection system for knowledge workers.

1. IT and Technology

To maintain technicians’ long-term cognitive attention and intrinsic motivation, tech firms should prioritize instrumental psychological resources. Complete technical tools, quiet workspaces, and independent decision rights can reduce attention fragmentation. Technical ranking and IP rewards can deliver emotional affirmation to sustain dedication.

1. R&D

Continuous creative research consumes cognitive resources rapidly; targeted psychological interventions are required to protect researchers’ concentration and motivation. Stable research funding and flexible schedules cut administrative distractions, while patent and milestone honors strengthen intrinsic work dedication.

1. Finance

Frequent compliance and data tasks interrupt financial staff’s cognitive focus, calling for systematic psychological resource maintenance. Unified analysis platforms streamline repetitive work to reserve cognitive capacity, and competence-oriented evaluation offers lasting value recognition against motivational burnout.

1. Consulting

Frequent cross-industry projects drain consultants’ psychological resources, requiring matched instrumental and emotional support. Industry databases and cross-departmental authority lower cognitive burden for scheme design, and project contribution rewards stabilize intrinsic motivation.

### 5.5 Limitations

Three key design limitations restrict interpretation of these correlational findings. First, the cross-sectional single-wave data only capture concurrent associations, making it impossible to verify temporal sequences or directional relationships; reverse or mutual correlations cannot be excluded, requiring multiwave longitudinal research to test hypothesized causal paths. Second, convenience sampling over-represents workers under 35, limiting the generalizability of the results to senior staff and traditional knowledge industries. Third, self-report surveys may trigger social desirability bias and subjective measurement error, distorting pairwise correlation magnitudes.

## 6 Conclusion

This study develops a multipath analytical model to examine the correlational relationships between two dimensions of organizational support (work support and value recognition) and job performance among Chinese knowledge workers, with dedication and concentration functioning as parallel partial correlational mediators based on cross-sectional sample data. The empirical results reveal that both types of organizational support are positively correlated with employee job performance, and work support exhibits stronger statistical associations with workers’ motivational and cognitive engagement states. By differentiating instrumental and socio-emotional organizational support and distinguishing motivational dedication from cognitive concentration, this study refines prior unified theoretical frameworks and adds localized empirical evidence to contextualize POS and work engagement research in the knowledge work context. The observed correlational patterns offer tentative, differentiated managerial implications for knowledge-intensive firms to optimize talent management and facilitate employee performance.

## Data Availability

The original contributions presented in the study are included in the article/supplementary material, further inquiries can be directed to the corresponding author.
